# Alpine Ski Motion Characteristics in Slalom

**DOI:** 10.3389/fspor.2020.00025

**Published:** 2020-03-27

**Authors:** Robert C. Reid, Per Haugen, Matthias Gilgien, Ronald W. Kipp, Gerald Allen Smith

**Affiliations:** ^1^Department of Physical Performance, Norwegian School of Sport Sciences, Oslo, Norway; ^2^Alpine Skiing, Norwegian Ski Federation, Oslo, Norway; ^3^Independent Researcher, Squaw Valley, CA, United States; ^4^Colorado Mesa University, Grand Junction, CO, United States

**Keywords:** alpine skiing, alpine ski, ski characteristics, ski motion, ski-snow interaction, ski mechanics

## Abstract

Important insight into ski function, and ultimately skier technique and tactics, can be gained by studying how measured ski trajectories compare to predictions based on theoretical models of ski-snow interaction mechanics. The aim of this investigation was to use a 3D kinematic data set collected on highly-skilled skiers during slalom race simulations to quantify ski motion characteristics and to compare these measures with theoretical predictions based primarily on ski geometrical characteristics. For slalom turns on moderate steepness (19°), ski edging angles reached maximum values of 65.7 ± 1.7° and 71.0 ± 1.9° for 10 and 13 m gate spacings. Turn radii reached minimum values of 3.96 ± 0.23 and 4.94 ± 0.59 m for the 10 and 13 m courses. These values were in good agreement with theoretical predictions by Howe (2001) of turn radius based on edging angle. Other results of the study support recent developments in understanding of the role which the ski shovel plays in groove formation during carving, and also point to the need for further study of how ski geometrical and physical characteristics interact to determine the ski's trajectory, particularly at low edge angles. These results have important implications for understanding the consequences that ski design can have for skier technique and tactics in competitive slalom skiing.

## Introduction

Turning technique is undoubtedly an important performance variable in alpine ski racing as can readily be ascertained by the attention it receives from coaches and athletes as well as from the sheer volume of scientific, professional, and lay publications addressing the topic. To turn, a skier manipulates the orientation and loading pattern of skis to generate a reaction force from the snow surface that allows redirection of trajectory and regulation of speed. Grasping the mechanics of how the ski interacts with the snow surface thus lays the foundation for understanding skier actions. Equally important, enhancing knowledge of ski-snow interaction mechanics is essential for the development of appropriate competition equipment regulations (Spörri et al., 2016a) to reduce the high injury rates seen in alpine ski racing (Florenes et al., 2009, 2012; Haaland et al., 2015). Theoretical models of ski-snow interaction mechanics have been described and tested using numerical simulations and physical models. However, there is a lack of empirical evidence validating these models under competitive conditions. And while several studies have investigated the effect of changes in ski geometry on injury risk, they have considered the athlete as a point mass (Gilgien et al., 2013, 2015c), relating equipment characteristics to gross biomechanical variables (i.e., speed, forces, trajectory) rather than the ski-snow interaction itself (Gilgien et al., 2016, 2018; Kröll et al., 2016a,b). To further our understanding of how ski characteristics influence the ski-snow interaction, the aim of this investigation was to use a 3D kinematic data set collected on highly-skilled skiers during slalom race simulations to quantify ski motion characteristics and to compare these measures with theoretical predictions.

## Literature Review

### Alpine Ski Characteristics

Alpine skis have geometrical and physical properties which influence how they interact with the snow surface. They have smooth, curved edge profiles referred to as sidecut, the amount of which varies depending on the type of ski. Two parameters are used to describe a ski's sidecut: Side camber and sidecut radius. Side camber (*SC*) is the distance between the ski at the narrowest part (waist) and a straight line between the widest points at the tail and shovel (Hirano and Tada, 1996; Kaps et al., 2001; Lind and Sanders, 2004; Federolf et al., 2010b). The sidecut radius (*R*_*SC*_) refers to the radius of a circle that intersects the side of the ski at the shovel, waist, and tail points while the ski is pressed flat on a planar surface (Kaps et al., 2001; Lind and Sanders, 2004). Primarily a function of the ski's width, thickness, and the materials used in its construction, a ski's flexural stiffness varies along its length (Howe, 2001; Lind and Sanders, 2004; Federolf et al., 2010b). The ski is in addition pre-stressed during construction as its layers are glued together causing the unloaded ski to take on a bent shape that is referred to as camber (Howe, 2001; Lind and Sanders, 2004; Federolf et al., 2010b). Together with the flexural stiffness distribution, the ski's camber affects the distribution of pressure under the ski's running surface when it is loaded. Torsional stiffness refers to the ski's ability to resist deformation about its longitudinal axis (Howe, 2001; Lind and Sanders, 2004) and, together with flexural stiffness, plays an important role in determining how aggressively the ski tip and tail interact with the snow when the ski is edged and loaded (LeMaster, 1999; Zorko et al., 2015).

### Ski Reference Systems

**Figure 1:**
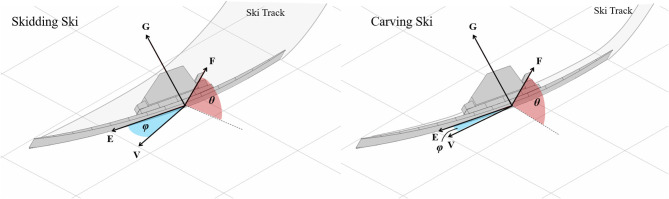
The ski edge angle (θ) and attack angle (ϕ) as defined by Lieu and Mote (1985). θ is the edge angle between the plane of the local snow surface and the running surface of the ski. The ski's angle of attack (ϕ) is the angle between the ski's longitudinal axis (E) and the center point's velocity vector (V) projected to a plane parallel to the local snow surface. The left panel presents a skidding ski with a relatively large attack angle, scraping a wide track into the snow surface. For contrast, the right panel presents a carving ski with a small angle of attack, leaving a narrow track in the snow.

To understand a ski's function, it is important to quantify its motion and orientation relative to the snow surface. Toward this end, Lieu (1982) and Lieu and Mote (1985) introduced a reference system to quantify a ski's orientation and the resulting angles with the snow surface (Figure 1). Originating at the ski center point, the **EFG** coordinate system defines the ski's position and orientation. **E** is oriented parallel to the ski's longitudinal axis, while **F** and **G** are directed lateral and normal to the ski sole surface, respectively.

Two angles between the ski and the snow surface are of particular importance to the ski's function. θ is the “edge angle” between the plane of the local snow surface and the running surface of the ski and describes to what degree the ski is tilted “on edge” relative to the local snow surface. The ski's “attack angle” (ϕ) is the angle between the ski's longitudinal axis **E** and the center point's velocity vector **V** in a plane parallel to the local snow surface. The attack angle describes to what degree the ski's longitudinal axis is oriented along it's direction of motion, an important factor influencing the nature of the ski-snow interaction. While ϕ represents the whole ski angle of attack, the local angle of attack at each position along the ski's length varies according to the ski's geometrical properties, its deformed shape under edging and loading, and its rotational and translational motion relative to the snow surface (Hirano and Tada, 1996; LeMaster, 1999; Tada and Hirano, 2002; Hirano, 2006; Spörri et al., 2016b). There are typically larger local attack angles on the ski forebody than on the ski afterbody, a fact that plays an important role in the ski's turning behavior.

### Skidding and Carving

When describing a ski's motion along the snow surface, two processes are generally recognized. During carving, a point along the ski's edge follows in the path of proceeding ski segments with minimal or no lateral displacement relative to the track (Lieu, 1982; Lieu and Mote, 1985; Brown and Outwater, 1989; Renshaw and Mote, 1989). In contrast, a ski that is sliding sideways across the snow surface as it moves forward is said to be skidding (LeMaster, 1999). A point on the ski's edge that is skidding does not follow in the path of proceeding points but rather shears through new snow as it moves across the snow surface (Lieu, 1982; Lieu and Mote, 1985; Brown and Outwater, 1989; Renshaw and Mote, 1989). In practitioner terms, an entire ski is often described as either skidding or carving. However, such a classification is an oversimplification as both carving and skidding may occur at the same time along different segments of the ski's length.

Lieu (Lieu, 1982) and Lieu and Mote (1985) modeled numerically the motion of skis through constant radius, constant speed turns and studied the effect of decreasing the ski angle of attack on ski motion. They found that at attack angles of ~11 degrees and greater, all points along the ski's length were in a skid mode. As the angle of attack was lowered to below 9 degrees, Lieu and Mote found that carving initiated at the tail of the ski. Further decreases in attack angle were associated with increased portions of the ski afterbody transitioning to carving. However, even in advanced carving stages, Lieu and Mote found that carving was limited to the ski's afterbody.

### Carving and Groove Formation

Lieu and Mote's (1985) findings are important in that they help to explain the mechanics of how a carving ski forms the groove in which the afterbody of the ski will ride. As the tip of an edged and loaded ski passes over a point on the snow surface, the first portion of the ski to contact the snow is often relatively soft in torsion and flexion and not heavily loaded. Accordingly, this portion of the ski may not penetrate the snow, but instead skid across the surface, vibrating in both flexion and torsion. With each passing point of the ski, stiffer portions of the forebody meet the snow and eventually enough pressure develops to push the ski into the snow surface. From this point on, the ski continues to penetrate deeper into the snow with each subsequent passing point, generating a groove (Tatsuno et al., 2009; Federolf et al., 2010b; Heinrich et al., 2010). The rising pressure increases the penetration depth and progressively compresses snow into the groove sidewall, both of which improve the groove's resistance to shear in preparation for the high forces which will occur as the boot passes (Mössner et al., 2006; Tatsuno et al., 2009). From the point of maximal pressure, the remainder of the ski is relatively unloaded in penetration and rides in the groove generated by the passage of the forebody. Seen in this way, the ski forebody does not ever carve—in a very strict sense of the word—since points along the forebody edge will trace their own trajectory, cutting new snow in the process, as has been predicted in both the research literature (Lieu, 1982; Lieu and Mote, 1985; Sahashi and Ichino, 1998; Casolo and Lorenzi, 2001) and practitioner textbooks (Joubert, 1980).

### Ski Trajectory

Early attempts to model the carving ski's trajectory were based solely on the geometrical properties of the ski and the resulting shape of the deformed ski as it is edged and loaded onto the snow surface. For rigid, planar snow surfaces, Howe (2001) proposed Equation 1 that relates the deformed ski's radius of curvature (*R*_*T*_) to its edge angle (θ) and sidecut radius (*R*_*SC*_):

(1) $$\begin{array}{l} R_{T}=R_{SC}\text{ cos}\theta \end{array}$$

Increasing the degree to which the ski is deformed onto the snow surface is expected to reduce *R*_*T*_, tightening the ski's turn trajectory. As Equation 1 suggests, one way of doing this is to increase the edging angle. As the ski is turned more onto edge, it will need to bend more to come into contact with the snow surface resulting in greater deformation and a shorter effective turn radius. This phenomenon has been demonstrated in a number of studies (e.g., Heinririch et al., 2006; Federolf et al., 2010a; Mossner et al., 2010). Along similar lines, increasing the ski's sidecut has also been found to amplify the ski's bending deformation, resulting in a decreased *R*_*T*_ (Hirano and Tada, 1996).

Despite this empirical evidence, Equation 1 is an oversimplification in several important ways. First, while the snow surface may at times be very hard, it is in reality never perfectly rigid. As previously described, skis penetrate into the snow surface, the depth of which is dependent upon the loading force, the snow's resistance to penetration, and the edging angle (Lieu and Mote, 1985; Brown and Outwater, 1989; Tada and Hirano, 2002; Federolf, 2005). This increases the ski's deformation and should therefore reduce *R*_*T*_ to a value lower than that estimated by Equation 1 (Howe, 2001; Kaps et al., 2001). This lead Howe to propose Equation 2 to account for non-rigid snow surfaces where *C* is the contact length, *SC* is the side camber, and *D*_*P*_ is the penetration depth:

(2) $$\begin{array}{l} R_{T}=\frac{C^{2}}{8\left[\left(SC/\text{cos}\theta \right)+D_{P}\text{ sin}\theta \right]} \end{array}$$

A second limitation of both Equations 1 and 2 is that they are based on the assumption that the entire length of the ski edge is in contact with the snow and carving. In reality however, certain portions of the ski will often alternate between carving and skidding modes depending on the balance between the local running surface pressure, the local edge angle and the local snow's shear strength.

Several researchers have recently reported experimental evidence indicating that carving skis do not follow exactly in the trajectory defined by the shape of the deflected edge on the snow surface, as both Equations 1 and 2 assume. In a study of elite skiers in giant slalom, Wimmer (2001) found only modest correlations (*r* = 0.39–0.57) between ski turn radius, as derived from reconstructed ski trajectories, and that calculated using Equation 1. He reported particularly large differences between reconstructed and predicted turn radii around turn transitions where the actual ski turn radius approached large values and the calculated turn radius approached a limit of *R*_*SC*_.

Kagawa et al. (2009), Tatsuno et al. (2009), and Yoneyama et al. (2008) measured ski deformations in carved turns using instrumented skis. Although they did not measure the ski's trajectory, they estimated that the actual ski turn radius was approximately twice that of the radius defined by the deformed ski edge. This they related to the mechanics of groove formation during carving and the idea that the ski forebody does not carve as it plows through the snow, establishing a groove.

Federolf (2005) and Federolf et al. (2010b) geodetically surveyed the track left in the snow by a carving ski in a giant slalom turn and compared the ski's actual turn radius to predictions using Howe's (2001) equation that accounts for snow penetration (Equation 2). He found that predicted turn radii based on the expected shape of the deformed ski underestimated actual measures and showed how the forebody of the ski will be deformed to a greater extent than can be accounted for in a carving ski's trajectory, particularly at higher edge angles. Using a Finite Element simulation of a carving ski that incorporated the mechanics of groove formation, Federolf et al. (2010a) found that Howe's equation agreed well with simulation results for low edge angles (<40 degrees) but that at high edge angles Howe's equation underestimated the ski's simulated turn radius.

That the carving ski's trajectory does not necessarily correspond to its deformed shape on the snow surface challenges our understanding—as both researchers and practitioners—of how the ski interacts with the snow surface. The purpose of this investigation was therefore to determine how well ski motion characteristics, which were measured in a previous kinematic study of skier technique, correspond to predictions of ski motion based on our theoretical understanding of ski snow interaction mechanics. In particular, our aims were to (1) examine how well-measures of local ski attack angles corresponded to Lieu and Mote's (Lieu, 1982; Lieu and Mote, 1985) prediction that carving is limited to the aft portion of the ski and (2) determine how well Howe's (2001) equation for turn radius based on ski geometry and edge angle (Equation 1) predicts actual ski trajectory measures.

## Methods

Six male members of the Norwegian national team (aged 17–20) volunteered to participate in a kinematic study of skier technique in April, 2006 (Reid et al., 2009; Reid, 2010; Federolf et al., 2012). This study was conducted in accordance with the Declaration of Helsinki and Norwegian law and was approved by the Norwegian Center for Research Data. All subjects gave their written informed consent prior to participation.

**Figure 2:**
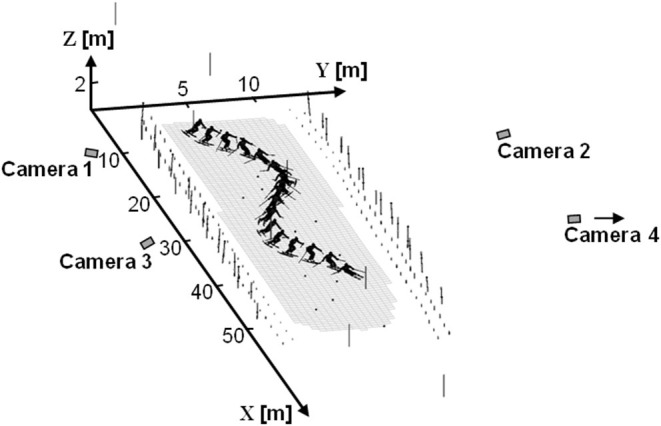
A graphical reconstruction of the experimental set-up. Control point positions are indicated by the small points and poles. Note that camera 4 was actually placed 30 m further to the right as seen from this perspective.

Skier kinematics were captured over two complete turns during slalom race simulations using a DLT-based method and four panning cameras (50 Hz) (Reid et al., 2009; Reid, 2010; Federolf et al., 2012). Skiers completed three runs on each of two courses set rhythmically with 10 and 13 m linear gate distances on even, moderately steep terrain (19° slope) and hard, compact snow conditions. The fastest run from each course was selected for further analysis giving a total of 24 analyzed turns for this investigation, 12 on each course. Two hundred and eight control points were positioned so as to surround the two turns of interest, creating a calibration volume of ~50 × 10 × 2 m (Figure 2). The control points, gates, and snow surface were geodetically surveyed using a theodolite. Camera images were individually calibrated using an average of 29 control points per frame and were synchronized after recording using an adaptation of the software genlock method (Pourcelot et al., 2000) that accommodates panning cameras. The ski tip (*TIP*), tail (*TAIL*), and ankle joint center (*AJC*) were manually digitized and reconstructed position data were filtered using a zero-lag, 2nd order, low-pass Butterworth filter and 20 padding points. The Challis residual autocorrelation algorithm (Challis, 1999) was used to individually determine the appropriate cut-off frequencies for each point (*TIP*, 9 Hz; *TAIL*, 8 Hz; *AJC*, 9 Hz).

One limitation of this approach is the error associated with manual digitization. Several measures were therefore taken to minimize digitization error including an extensive training program with feedback; the use of photographs of equipment to assist point identification; and the identification of outliers in the data set for double-checking and correction. Measurement accuracy was assessed using control points positioned on the snow surface close to the skier's trajectory but which were removed from the calibration sequence for the purpose of accuracy assessment. A total of 980 so-called “*non*-control point” reconstructions were assessed across all 12 of the analyzed trials. *Non*-control point root mean squared error (RMSE) was 4, 5, and 2 mm in the **X**, **Y**, and **Z** dimensions, respectively. Pooled standard deviations of segment lengths were used to assess digitizer reliability. Over the 12 analyzed trials, the skis were reconstructed 2,170 times with a pooled standard deviation for the ski running surface length of 11 mm.

**Figure 3:**
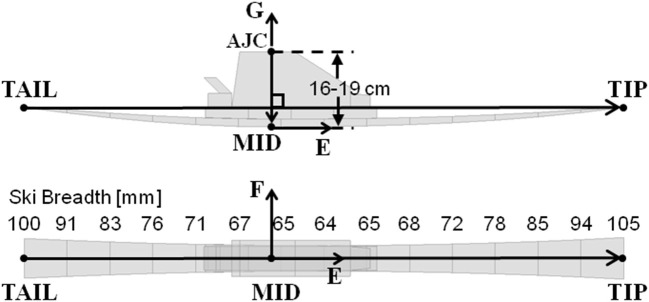
The 15 segment ski model fitted to *TIP, TAIL*, and *AJC*. *MID* was defined as the point along the ski sole 16–19 cm below *AJC*, in the direction perpendicular to the *TIP*-*TAIL* vector.

The *TIP, TAIL*, and *AJC* position data were fit with a 15 segment model of a 14 m sidecut radius ski. To accomplish this, a third point on the ski sole (*MID*) was defined as the point between 16 and 19 cm below *AJC* in the direction perpendicular to the *TIP*–*TAIL* vector, assuming that the ski sole to foot sole distance was close to the maximum allowable in competition (10 cm in 2006) and that the foot sole to *AJC* distance was between 6 and 9 cm (Figure 3). The actual distance was chosen for each athlete individually so as to obtain 0 mm ski flexural deformation at turn transitions. Subsequent to determining *MID*, the ski midline was then approximated by fitting *TIP, MID*, and *TAIL* with a cubic spline function, constructing points at 15 evenly spaced intervals. Positions along the ski's edges were then approximated using the average sidecut profile of 11 slalom skis. The reconstructed ski running surface length had a pooled standard deviation of 11 mm (*n* = 2,170 measurements taken over 12 trials).

In order to calculate ski motion characteristics, a smooth snow surface model with continuous first- and second-order derivatives was generated based on the Delaunay triangulation of the geodetically captured snow points (Gilgien et al., 2015a,b). The ski edge angle (θ) was defined in accordance with Lieu (1982) as the angle between the plane of the local snow surface and the running surface of the ski. θ is however probably most appropriately described as a rough estimate of the ski edge angle. The actual edge angle can be expected to differ somewhat from this estimate depending on the individual's binding and boot set-up (Müller et al., 1998). In addition, the edge angle is likely to vary along the ski's length due to ski flexion and torsion deformations whose measurement was beyond the resolution of the method employed in this investigation. Complicating matters further is the fact that the exact nature of the local snow surface was not precisely known and can be expected to progressively change with each passing skier as the snow is scraped and deformed.

The ski attack angle (ϕ), defined as the angle between the ski's longitudinal axis and the center point's velocity vector (Lieu, 1982), was quantified to describe the degree of skidding and carving. Local ski attack angles ϕ_*E*_ for points along the outside ski's interacting edge were calculated in a similar manner for comparison with Lieu and Mote's predictions of ski motion (Lieu, 1982; Lieu and Mote, 1985).

The radius of curvature of the ski center point's trajectory (*R*_*SKI*_) at time point index *i*, parallel to the least squares plane of the snow surface, was calculated by determining the radius of the circle fitting the center point's positions at time point index *i, i*−3, and *i* + 3. As the actual penetration depth was not measured, the simpler Howe (2001) equation (Equation 1) was used to predict turn radius (*R*_*HOWE*_) based on the ski's sidecut radius and the measured edge angle. These theoretical turn radii were compared with those directly measured during slalom turns on each course.

## Results

**Figure 4:**
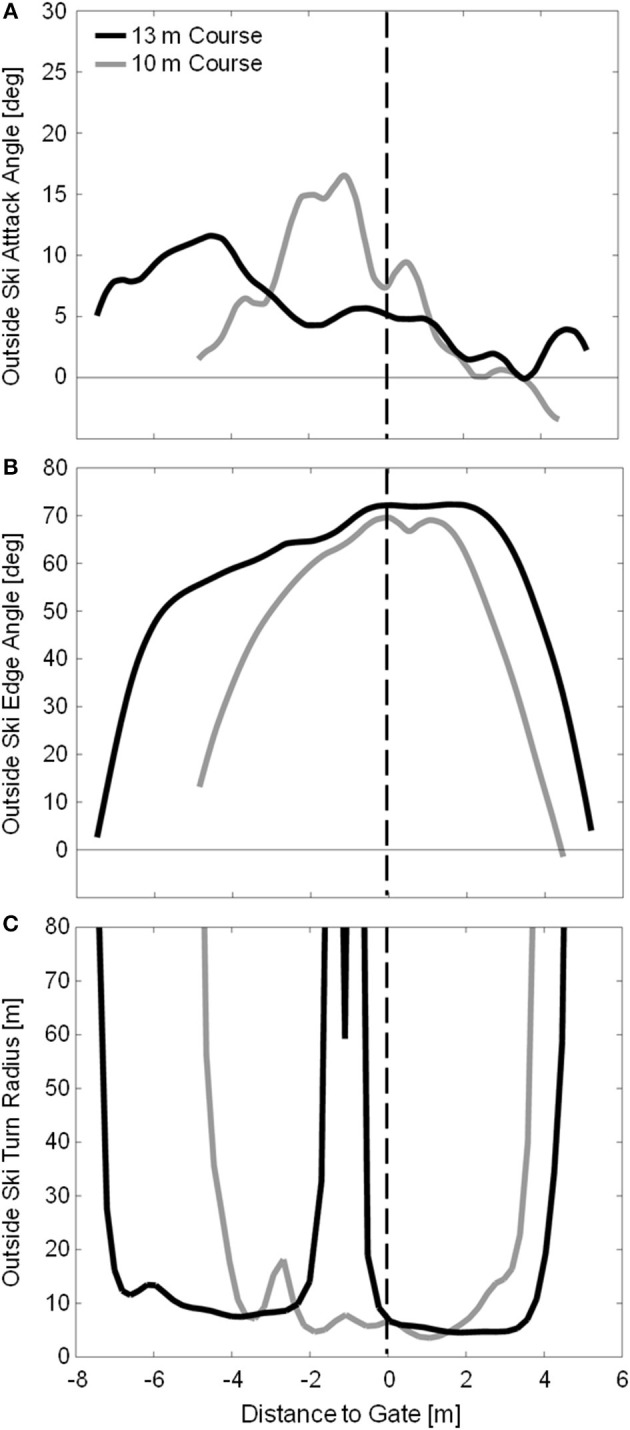
Measured outside ski attack angle **(A)**, edge angle **(B)**, and turn radius **(C)** for sample turns from the 10 and 13 m courses in gray and black, respectively. Due to the different course setting, the data from the two courses are coordinated using the gate as a common point, and presenting the X axis as distance to gate. Gate passage is indicated by the vertical dashed line. It is relevant to note that turns on the 13 m course start much higher up on the slope relative to the gate than on the 10 m course.

Figure 4 shows the outside ski attack angle, edge angle, and turn radius for sample turns on the 10 and 13 m courses. At the start of the turn cycle, the new outside ski was already slightly edged to on average 5.1 ± 4.6 and 4.5 ± 5.1 degrees on the 10 and 13 m courses, respectively. On the 10 m course, edge angle progressively increased through the first half of the turn, reaching an average maximum angle of 65.7 ± 1.7 degrees just after gate passage. On the 13 m course, there was an initial rapid rise in edge angle followed by a period of more gradual increase, reaching maximum angles of 70.2 ± 1.3 degrees at approximately gate passage. Edge angle then declined rapidly during turn completion for both gate distances.

The outside ski had on average a positive attack angle of 3.1 ± 2.4 and 0.5 ± 2.4 degrees at the transition between turns on the 10 and 13 m courses, respectively, indicating that the skis were already being oriented for the upcoming turn during the completion of the previous turn. Attack angles rose rapidly during turn initiation, reaching average maximums of 15.1 ± 5.3 and 12.1 ± 4.9 degrees early in the turn for the 10 and 13 m courses, respectively. During the first half of the turn cycle, attack angles were greater on the 10 m course, in particular from 10 to 45 % of the turn cycle, indicating that there was a greater degree of skidding used on the 10 m course, on average. There was, however, a substantial amount of individual variation on both courses during this part of the turn with some turns being carved and some skidded. The outside ski then shifted to carving by about gate passage with all turns on both courses being completed at attack angles below 4 degrees.

To allow comparison with Lieu and Mote's predictions (Lieu, 1982; Lieu and Mote, 1985), Figure 5 presents the local ski attack angle (ϕ_*E*_) data averaged according to position along the ski's longitudinal axis and whole ski attack angle (ϕ) for the steering phase of the turn cycle. To help visualize the meaning of the local attack angle data, sample graphics were generated showing ski edge point trajectories during the transition from skidding to carving. The dashed and solid lines indicate ski forebody and rearbody point trajectories, respectively.

**Figure 5:**
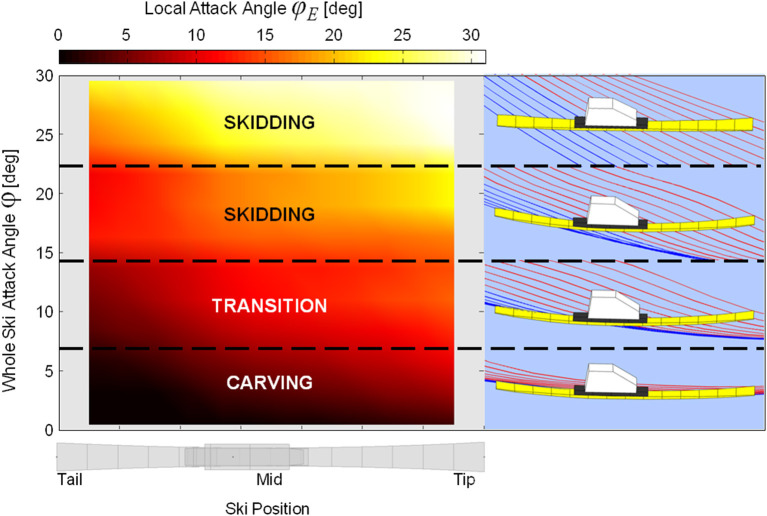
Mean local ski attack angle averaged across whole ski attack angle (left panel). An example ski making the transition from skidding to carving through a turn is shown in the right panels.

**Figure 6:**
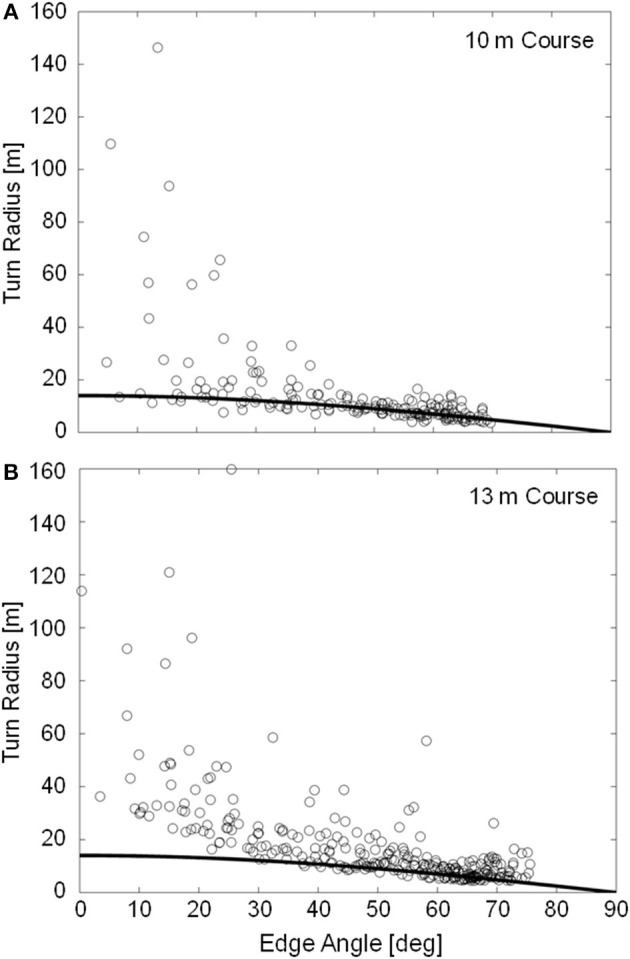
Instantaneous measured outside ski turn radius (R_T_, data points) and predicted outside ski turn radius using Equation 1 (R_HOWE_, data line) for the 12 analyzed trials on the 10 m **(A)** and 13 m **(B)** courses. Data are limited to time points where the ski was carving (ϕ < 5°, *n* = 185 and 298 for the 10 and 13 m courses, respectively).

Minimum outside ski turn radius measurements were slightly longer on the 13 m course (4.94 ± 0.59 m) than on the 10 m course (3.96 ± 0.23 m) despite the higher maximum edge angles observed on the 13 m course. In contrast to the 10 m course, large fluctuations in *R*_*SKI*_ were observed during the early to mid-portion of the turn on the 13 m course, as exemplified in Figure 4C. Figure 6 compares the measured turn radius (*R*_*SKI*_) to that predicted using Howe's (2001) equation (*R*_*HOWE*_) for time points where the ski was considered to be carving, defined as ϕ < 5 degrees. RMSE between the measured (*R*_*SKI*_) and predicted (*R*_*HOWE*_) turn radii was 27.2 and 44.5 m for the 10 and 13 m courses, respectively. However, prediction error was much higher for edge angles below 45 degrees (42.0 and 71.5 m RMSE for the 10 and 13 m courses, respectively) than for edge angles above 45 degrees (2.5 and 6.4 m RMSE for the 10 and 13 m courses, respectively).

## Discussion

### Skidding and Carving

There was a slightly greater degree of skidding on the 10 m course, primarily in the first portion of the turn. However, with average maximum attack angles of 15 and 12 degrees seen on the 10 and 13 m courses, respectively, the skidding in this study is perhaps best described as moderate compared to what can often be observed in typical competition conditions. That skiers used some skidding in this investigation is not surprising considering that the experimental set-up was on moderately steep terrain where skidding can be used to regulate speed.

In the comparison with Lieu and Mote's predictions (Lieu, 1982; Lieu and Mote, 1985) (Figure 5), some variability in local ski attack angle patterns was evident, likely due to variation in the mechanical and geometrical properties of the skis used by the athletes as well as irregularities in the ski's motion. In general, however, local attack angles were high along the entire ski when whole ski attack angles were greater than about 15 degrees, indicating that skidding processes dominated. Below this level, local attack angles in the aft-most ski segments reduced while those of the forebody segments remained elevated. Local attack angles of the aft-most segments reached 2 to 5 degrees as whole ski attack angles approached 8 degrees, indicating that these points began carving, in good accordance with Lieu and Mote's results. Further decreases in the whole ski attack angle were associated with increasing numbers of tail segments carving, along with the reduction of forebody segment attack angles. The ski reached an advanced carving stage at whole ski attack angles of ~3 degrees, although local forebody segment attack angles remained slightly elevated, indicating that this part of the ski was still machining new snow, also in good accordance with Lieu and Mote's work as well as Tatsuno's (2009), Federolf's (2005), and Federolf et al. (2010b) descriptions of ski shovel function.

### Ski Trajectory

The outside ski experienced high intensity turning over the majority of the turn cycle, in some instances starting prior to the transition between turns. For portions of the turn cycle where the ski was carving and the edge angle was relatively high (θ > 45 degrees, see Figure 6), Howe's equation (Equation 1) performed surprisingly well in predicting the actual ski turn radius, considering the simplicity of the equation and the complex interaction of variables influencing the ski-snow interaction. This relatively strong association between Howe's model and measured data seems to indicate how important ski geometric properties—in particular the sidecut radius—are in determining a ski's behavior on snow during carved turns at high edge angles. At low edge angles, however, Howe's equation greatly underestimated the actual turn radius. This contrasts with earlier studies (Federolf, 2005; Federolf et al., 2010a,b) where it was found that Howe's equations performed better at low edge angles and systematically underestimated the actual turn radius at edge angles higher than ~45 degrees. In the current investigation, it was not until edge angles reached over 70 degrees that *R*_*HOWE*_ appeared to underestimate *R*_*SKI*_ (on the 13 m course). One possibility for this contrast in results may be that the current investigation was conducted on a relatively hard snow surface where penetration depths were limited such that the ski's deformation more closely matched the shape of the groove being generated in the snow and the ski's trajectory.

There were, nevertheless, two situations in which Howe's Equation 1 failed to capture the ski's trajectory. First, *R*_*SKI*_ and *R*_*HOWE*_ differed substantially during the transition between turns where *R*_*SKI*_ approached infinity and *R*_*HOWE*_ approached a limit of *R*_*SC*_, similar to Wimmer's (2001) findings. That the ski can carve at turn radii much longer than that predicted by Equation 1 for low edge angles may be explained to a certain extent by the ski's physical properties. Torsional stiffness plays an important role as the ski shovel and tail twist under the moments generated during their interaction with the snow. If the resulting torsional deformations are large enough to reduce the ski's local edge angle below a certain threshold, that portion of the ski will disengage from the snow and begin to skid or lose contact with the snow entirely. LeMaster (1999) explained that at low edge angles this phenomenon may reduce the engaged, carving section of the ski to the middle portion that has less sidecut, in effect decreasing the ski's turn radius. If this holds true, then the ski's physical properties, including its flexural and torsional stiffness distributions, are important parameters which affect the carving ski's trajectory at low edge angles.

**Figure 7:**
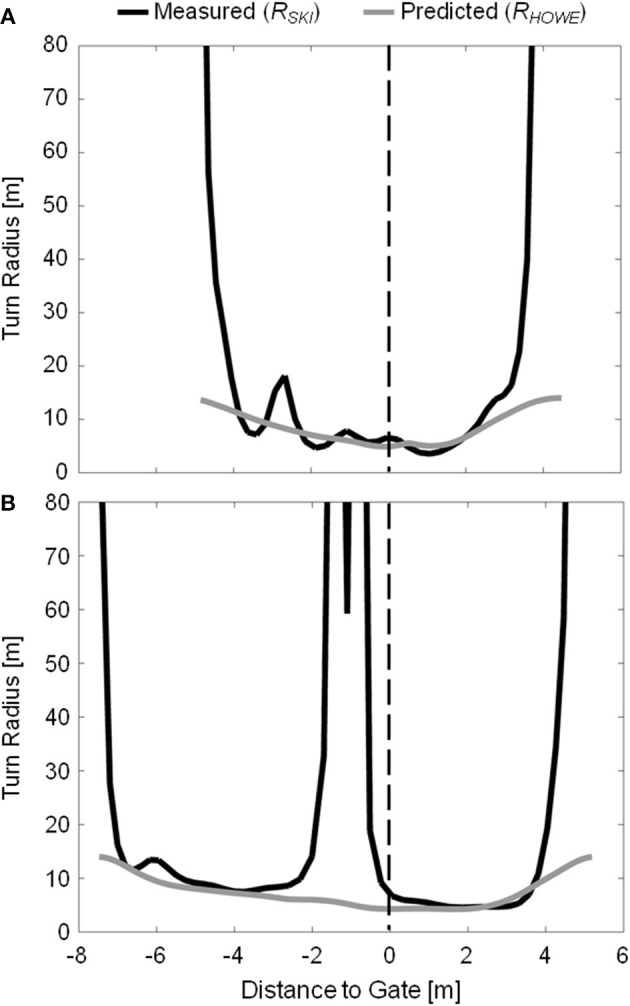
Measured (*R*_*SKI*_, dark lines) and predicted (*R*_*HOWE*_, gray lines) outside ski turn radius for sample turns on the 10 m **(A)** and 13 m **(B)** courses. Due to the different course setting, the data from the two courses are coordinated using the gate as a common point, and presenting the X axis as distance to gate. The vertical dashed line indicates gate passage.

Howe's Equation 1 also did not capture well the large, intermittent fluctuations in *R*_*SKI*_ that were apparent, particularly on the 13 m course (Figure 7). That these disturbances in ski trajectory did not occur to the same degree on the 10 m course seems counter-intuitive knowing that there was a greater degree of skidding on the 10 m course and suggests that somehow the mechanism may be associated with carving mechanics. This result is perhaps particularly striking considering that other researchers have also observed possibly related phenomena when studying carved turns. Of particular note, Federolf (2005) and Federolf et al. (2010b) observed times where the outside ski reduced turning in the first portion of the turn in his kinematic analysis of carving ski trajectories in giant slalom which they attributed to lateral drifting. In their comparison of an athlete skiing on carving and conventional equipment, Raschner and colleagues (Raschner et al., 2001; Müller and Schwameder, 2003) reported irregular force-time curves when skiing on the carving equipment, an unexpected finding that they also attributed to repeated lateral skidding.

**Figure 8:**
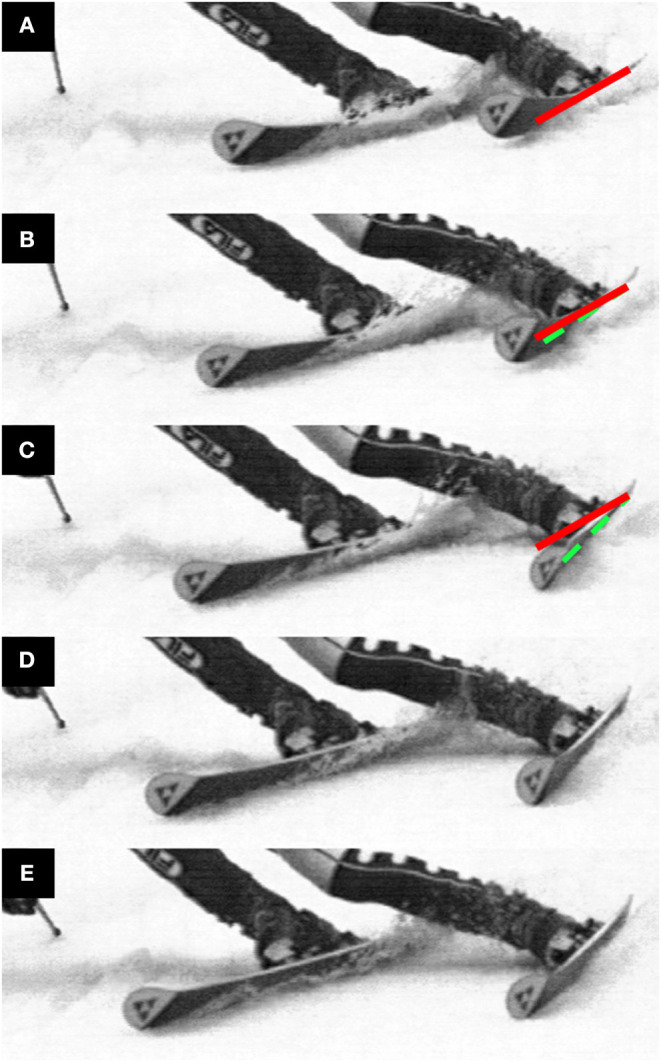
High-speed video footage of a carving ski undergoing a disturbance possibly similar to that observed in the current investigation. This video, taken during the women's World Cup giant slalom at Åre in March, 2006, was filmed at 1,500 fps. To help the reader visualize the outside ski's motion, the solid, black line indicates the original ski orientation from Frame **(A)** while the dashed, white line indicates the changing ski's orientation. From Frame **(A–C)**, the ski shovel sways toward the outside of the turn. The shovel reaches and engages the snow surface in Frame **(C)**. Groove formation is then redirected onto a new trajectory in Frames **(D, E)** with the increased distance between the skier's feet indicating that the outside and inside skis have come onto diverging trajectories.

One obvious explanation for these occurrences could simply be that irregularities in the snow surface interfered with the ski's trajectory and resulted in drifting or skidding. This possibility cannot be ruled out in the current investigation. However, there are alternative explanations which we believe are more likely. The fact that these disturbances occurred to a greater extent on the 13 m course suggests that differences in carving and skidding ski-snow interaction mechanics may help explain their occurrence. One such important difference is the process of groove formation. When carving, the ski will be tilted slightly in the snow so that the foremost points on the ski are disengaged from the surface (Lieu and Mote, 1985). The relatively soft tip is then free to vibrate back and forth in flexion and torsion as the shovel digs the groove in which the remainder of the ski will follow. It may be that at times when the tip sways toward the outside of the turn, it catches and engages in the snow surface, consequently redirecting groove formation toward the outside of the turn and away from the skier. There is some observational evidence that this may be the case. An example of this phenomenon is shown in Figure 8 which shows a photo sequence generated from high-speed video taken during a women's World Cup giant slalom. This mechanism by which the ski may unexpectedly take a trajectory away from the skier could, in the most extreme cases, lead to potentially injurious situations such as the “slip-catch” and “dynamic snowplow” mechanisms described by Bere et al. (2011).

That all of the 12 analyzed turns on the 13 m course showed some form of disturbance just prior to gate passage suggests another, perhaps related mechanism. During the first half of the turn, outside ski trajectories on the 13 m course were much higher on the slope, relative to the approaching gate, than on the 10 m course. Moreover, skis were edged and turning much higher on the slope on the 13 m course (see Figure 4) while after gate passage the trajectories from both courses were similar. It may be that on the 13 m course, skis turned too much, too high on the slope relative to the approaching gate, and that the disturbances measured in ski turn radius were actually the result of having to reorient the ski onto a new trajectory to avoid skiing on the wrong side of the gate.

That the ski seems to be re-oriented suddenly, as opposed to gradually corrected over the entire first half of the turn, may indicate that the skier's control over the degree to which a carving ski turns for a given edge angle is more limited than traditionally thought. Taking this line of reasoning further, an explanation for why these disturbances did not occur on the 10 m course to the same extent as on the 13 m course may be that the ski's trajectory on the 10 m course more closely matched its physical and geometrical characteristics so that the skiers did not have to correct its trajectory during the turn.

## Conclusions and Further Perspectives

In summary, this study has captured ski motion characteristics during slalom race simulations and compared these measures with theoretical predictions of ski motion. During the transition from skidding, the tail of the ski initiated carving as the ski attack angle reduced below 8 degrees, in good accordance with Lieu and Mote's results (Lieu, 1982; Lieu and Mote, 1985). The ski reached an advanced carving stage at whole ski attack angles of ~3 degrees, although local attack angles along the ski forebody remained slightly elevated, also in good accordance with theoretical models of ski shovel function during carving (Lieu, 1982; Lieu and Mote, 1985; Yoneyama et al., 2008; Tatsuno et al., 2009; Federolf et al., 2010b).

Important insight into ski function can be gained by studying how measured ski trajectories compare to prediction models that are based on the shape of the deformed ski, such as Howe's models (Howe, 2001). In this investigation, Howe's equation (Equation 1) performed surprisingly well for edge angles above ~45 degrees indicating that ski geometry, in particular sidecut radius, is an important variable determining the ski's trajectory at high edge angles. On a practical level, these results suggest that the skier's trajectory will largely be determined by the ski sidecut radius in a carved turn at high edge angles. This understanding may have consequences for equipment design and course setting both with respect to performance and safety (Kröll et al., 2016a,b).

The results from this study were more complicated for lower edge angles, however. Howe's Equation 1 prediction accuracy progressively degraded with decreasing edge angles, which is in good agreement with some previous work (Wimmer, 2001) but in contrast with others (Federolf et al., 2010a). This suggests that variables other than sidecut radius alone influence the ski's trajectory at low edge angles, such as other ski physical properties or skier technique. Therefore, future investigations should consider how ski geometry, in combination with flexural and torsional stiffness distributions, determine the carving ski's trajectory on different types of snow conditions. This study has focused on carved turns. However, understanding how equipment characteristics influence skidded turns is equally important. Following this line of research to better understand how ski characteristics influence the ski-snow interaction can support the ski industry in developing equipment for improved performance, enjoyment and safety.

## Data Availability Statement

The datasets for this article are not publicly available due to intellectual property reasons. Requests to access the datasets should be directed to the corresponding author.

## Ethics Statement

The studies involving human participants were reviewed and approved by Ombudsman for Privacy in Research, Norwegian Social Science Data Services, AS. The patients/participants provided their written informed consent to participate in this study.

## Author Contributions

RR, PH, MG, and RK conducted the data collection. RR, MG, and GS conducted the analysis. All authors contributed to the writing, publication of the study, and design and scientific content of the study.

### Conflict of Interest

The authors declare that the research was conducted in the absence of any commercial or financial relationships that could be construed as a potential conflict of interest.
